# Current Perspective Regarding the Immunopathogenesis of Drug-Induced Hypersensitivity Syndrome/Drug Reaction with Eosinophilia and Systemic Symptoms (DIHS/DRESS)

**DOI:** 10.3390/ijms22042147

**Published:** 2021-02-21

**Authors:** Fumi Miyagawa, Hideo Asada

**Affiliations:** Department of Dermatology, Nara Medical University School of Medicine, Nara 634-8522, Japan; asadah@naramed-u.ac.jp

**Keywords:** severe cutaneous adverse reactions, drug-induced hypersensitivity syndrome, drug reaction with eosinophilia and systemic symptoms, herpesvirus, autoimmune disease, pathomechanism

## Abstract

Drug-induced hypersensitivity syndrome/drug reaction with eosinophilia and systemic symptoms (DIHS/DRESS) is a severe type of adverse drug eruption associated with multiorgan involvement and the reactivation of human herpesvirus 6, which arises after prolonged exposure to certain drugs. Typically, two waves of disease activity occur during the course of DIHS/DRESS; however, some patients experience multiple waves of exacerbation and remission of the disease. Severe complications, some of which are related to cytomegalovirus reactivation, can be fatal. DIHS/DRESS is distinct from other drug reactions, as it involves herpes virus reactivation and can lead to the subsequent development of autoimmune diseases. The association between herpesviruses and DIHS/DRESS is now well established, and DIHS/DRESS is considered to arise as a result of complex interactions between several herpesviruses and comprehensive immune responses, including drug-specific immune responses and antiviral immune responses, each of which may be mediated by distinct types of immune cells. It appears that both CD4 and CD8 T cells are involved in the pathogenesis of DIHS/DRESS but play distinct roles. CD4 T cells mainly initiate drug allergies in response to drug antigens, and then herpesvirus-specific CD8 T cells that target virus-infected cells emerge, resulting in tissue damage. Regulatory T-cell dynamics are also suggested to contribute to the diverse symptoms of DIHS/DRESS. However, the pathomechanisms of this complex disease remain largely unknown. In particular, how viral infections contribute to the pathogenesis of DIHS/DRESS and why autoimmune sequelae arise in DIHS/DRESS are yet to be elucidated. This review describes the clinical features of DIHS/DRESS, including the associated complications and sequelae, and discusses recent advances in our understanding of the immunopathogenic mechanisms of DIHS/DRESS.

## 1. Introduction

Severe cutaneous adverse reactions (SCARs) encompass a heterogenous group of delayed hypersensitivity reactions, most of which are caused by drugs. These include Stevens-Johnson syndrome (SJS), toxic epidermal necrolysis (TEN), drug-induced hypersensitivity syndrome/drug reaction with eosinophilia and systemic symptoms (DIHS/DRESS), and acute generalized exanthematous pustulosis. DIHS/DRESS is a potentially fatal multiorgan hypersensitivity reaction associated with the reactivation of human herpesvirus 6 (HHV-6) [1,2]. The term DRESS was first proposed in 1996 for a hypersensitivity syndrome that had previously been reported under various names, such as anticonvulsant hypersensitivity syndrome, allopurinol hypersensitivity syndrome, sulfone syndrome, and dapsone hypersensitivity, depending on the causative drug [3]. It should be noted that DIHS and DRESS were defined by the Japanese Research Committee on Severe Cutaneous Adverse Reactions (J-SCAR) [1] (Table 1) and the RegiSCAR group [2] (Table 2), respectively, but both diseases probably belong to the same disease spectrum [4,5]. Specifically, HHV-6 reactivation is included as one of the diagnostic criterion for DIHS, but not for DRESS, and typical DIHS may represent a severe form of DRESS [6].

**Figure 1:**
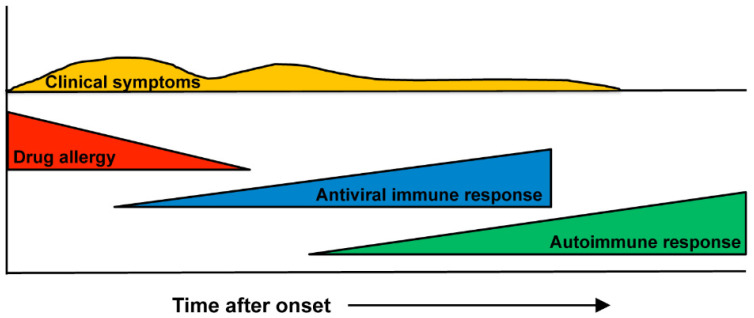
Scheme depicting the clinical course and immunological mechanism involved in DIHS/DRESS. DIHS/DRESS are considered to occur as a result of complex interactions between several herpesviruses and comprehensive immune responses, including drug-specific immune responses and antiviral immune responses. After the resolution of the disease, autoimmune responses may appear.

Our understanding of the pathophysiology of DIHS/DRESS has evolved considerably over the last decade. DIHS/DRESS is distinguished from other drug eruptions by several characteristics, including the fact that it is associated with a limited number of causative drugs, exhibits late onset and a prolonged course, and patients’ clinical symptoms deteriorate after treatment with the causative drug is discontinued. Herpesvirus reactivation, multiorgan involvement, a relapsing/remitting disease course, and delayed autoimmune diseases are also prominent features of DIHS/DRESS. The current consensus on the pathogenesis of DIHS/DRESS is that it occurs as a result of complex interactions between several herpesviruses and comprehensive immune responses, including drug-specific immune responses and antiviral immune responses, each of which may be mediated by distinct types of immune cells [7] (Figure 1). Although the association between herpesviruses and DIHS/DRESS is well established, several aspects of these conditions, including how viral infections contribute to the pathogenesis of DIHS/DRESS and why autoimmune sequelae arise in DIHS/DRESS are yet to be elucidated. In this review, we summarize the clinical features of DIHS/DRESS, including the associated complications and sequelae, and explore current knowledge regarding the immunopathogenic mechanisms of DIHS/DRESS, particularly the roles of T cells and herpesviruses.

## 2. Clinical Features

### 2.1. Clinical Symptoms

**Figure 2:**
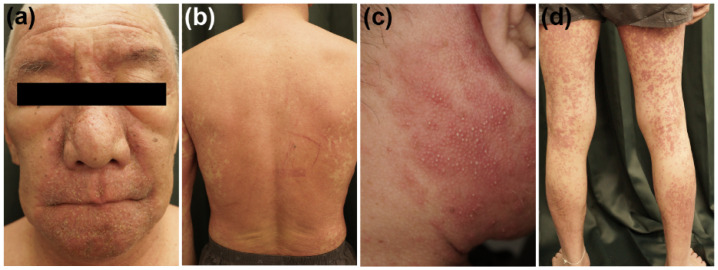
Clinical manifestations of DIHS/DRESS. (**a**) Facial erythema, scaling, and peri-orbital edema. (**b**) Diffuse erythematous rash on the back. (**c**) Pustules on the face. (**d**) Purpura on the legs.

DIHS/DRESS is characterized by a delayed onset, i.e., it usually occurs 3 weeks to 3 months after the initiation of treatment with the causative drug, and the deterioration of clinical symptoms after the cessation of such treatment [1,2,7]. These features are peculiar to DIHS/DRESS and are not seen in other types of drug eruptions. A limited number of drugs, including carbamazepine, phenytoin, phenobarbital, zonisamide, lamotrigine, mexiletine, dapsone, sulfasalazine, minocycline, allopurinol, and vancomycin, are implicated cause in most cases of DIHS/DRESS [4,7,8]. HHV-6 reactivation has also been demonstrated to be involved in DIHS/DRESS, and it generally occurs 2–3 weeks after the onset of rashes in DIHS/DRESS [9,10,11]. The symptoms of DIHS/DRESS include rash development associated with facial and periorbital edema (Figure 2a), lymphadenopathy, and fever [7]. DIHS/DRESS typically begins with a fever and maculopapular eruptions, which often generalize into severe exfoliative dermatitis or erythroderma (Figure 2b). Pinhead-sized pustules (Figure 2c), blisters, and purpura (Figure 2d) are occasionally present. There is usually no mucocutaneous involvement. The laboratory findings of DIHS/DRESS include leukocytosis, eosinophilia, atypical lymphocytosis, and liver abnormalities, which can vary in severity. Systemic involvement includes hepatitis and/or interstitial pneumonia. Interstitial nephritis or carditis may also be found. The typical clinical course of DIHS/DRESS involves a bimodal distribution of clinical symptoms, i.e., they are most severe at onset and 2–3 weeks after onset (at the time of HHV-6 reactivation). However, in severe cases symptoms can continue to deteriorate or several flare-ups can be seen, even weeks after the discontinuation of the causative drug. Some patients develop autoimmune diseases after the resolution of DIHS/DRESS (Figure 1). The mortality rate of DIHS/DRESS ranges from approximately 10–20% [7,8], and the risk of death is correlated with the degree of hepatic or renal involvement [7]. The complications and sequelae observed in DIHS/DRESS are detailed below.

### 2.2. Complications

The prognosis of DIHS/DRESS depends on how severe the associated complications are. Visceral organ involvement typically manifests as hepatitis, but may include lymphadenopathy, interstitial nephritis, interstitial pneumonitis, and/or myocarditis [8]. The most frequently affected organs are the liver (75%), kidneys (37%), and lungs (32%) [8]. Liver involvement, which is the most common visceral manifestation of DIHS/DRESS, can lead to fulminant hepatic failure and death. A higher risk of liver involvement appears to be seen in phenytoin- or dapsone-induced DIHS/DRESS [7]. Renal involvement is known to occur at a higher frequency in allopurinol-induced DIHS/DRESS [7] and can sometimes have a prognostic impact. Myocarditis is an under-recognized manifestation of DIHS/DRESS with a high mortality rate [12].

Some of the complications of DIHS/DRESS, such as pneumonia, hepatitis, and gastroenteritis, are known to be caused by cytomegalovirus (CMV) reactivation [13]. It is now largely accepted that DIHS/DRESS can be associated with CMV reactivation, which occurs 3 to 7 weeks after onset and can lead to a fatal outcome if the patient develops overt CMV disease [13,14]. Although most patients that experience CMV reactivation do not exhibit evidence of CMV disease, overt CMV disease, which manifests as skin ulcers, pneumonia, hepatitis, and/or gastroenteritis, is regarded as the most important factor determining the prognosis of DIHS/DRESS patients [14]. Because CMV reactivation has also been observed, even if less frequency, in SJS/TEN [15], CMV reactivation may be a complication of immunosuppressive therapy.

### 2.3. Sequelae (Autoimmune Disease)

The development of autoimmune diseases, such as autoimmune thyroiditis, type I diabetes mellitus, and autoimmune hemolytic anemia, can occur as a late complication of DIHS/DRESS, i.e., several months to years after the resolution of DIHS/DRESS [16,17]. The overall cumulative incidence of long-term sequelae in DIHS/DRESS has been reported to be 11.5% [16]. A study of the sequelae of 145 DIHS/DRESS patients conducted by the Asian Research Committee on Severe Cutaneous Adverse Reactions (ASCAR) revealed that the following autoimmune diseases can newly develop after the resolution of DIHS/DRESS: thyroid diseases, diabetes mellitus, systemic lupus erythematosus, arthritis, alopecia, and vitiligo [17]. The development of other autoimmune diseases, including sclerodermoid graft-versus-host disease-like lesions [18] and chronic inflammatory demyelinating polyneuropathy [19], has also been reported in sporadic case reports. The subsequent development of type III polyglandular autoimmune syndrome has also been reported in a 6-year-old boy who developed multiple autoimmune diseases, including thyroiditis, type I diabetes, alopecia, vitiligo, and uveitis due to Vogt-Koyanagi-Harada disease after the resolution of DIHS/DRESS [20]. The immunological mechanism underlying the development of autoimmune diseases in DIHS/DRESS currently remains unknown. A recent report suggested that higher plasma interferon (IFN)-γ-induced protein (IP)-10 levels are associated with the development of long-term sequelae in patients with DIHS/DRESS [21]. IP-10 has already been known to be associated with idiopathic autoimmune diseases, such as type 1 diabetes, thyroiditis, vitiligo, and alopecia areata [21].

## 3. Immunopathogenesis

### 3.1. Antigen Presentation

Although extensive studies have been performed, the pathogenesis of DIHS/DRESS is not yet fully understood. DIHS/DRESS is attributed to an immunological reaction to a drug or drug metabolites because its clinical symptoms, such as rashes and fever, reappear upon the re-administration of the causative drug. A positive lymphocyte transformation test (LTT) result for a suspected causative drug in a patient with DIHS/DRESS and the generation of a T-cell clone from a DIHS/DRESS patient that reacts to the suspected causative drug [22] are strongly indicative of a T-cell-mediated immune response against the drug. A positive LTT result also suggests that the relevant drug can directly interact with T-cell receptors (TCRs) without needing to undergo any prior metabolic processing or protein binding [22]. Thus, the pathogenesis of DIHS/DRESS is considered to involve T-cell-mediated delayed hypersensitivity reactions involving interactions between small-molecule drugs, human leukocyte antigens (HLAs), and TCRs, as is the case with most SCARs. Currently, three main models for the mechanisms responsible for T-cell-mediated SCARs have been discussed: the hapten/prohapten model, the pharmacological interactions of drugs with immune receptors (p-i) concept, and the altered peptide repertoire model [23]. In the hapten/prohapten model, drugs and their metabolites are considered to be too small to be immunogenic per se. This implies that a drug (e.g., penicillin) binds covalently to an endogenous protein (carrier), forming a hapten-carrier complex, which is processed by antigen-presenting cells and recognized by a TCR, and hence, a drug-specific immune response can occur [24]. The p-i concept, which was proposed by Pichler [25], states that the causative drug binds non-covalently to HLAs and/or TCRs, directly stimulating specific TCRs and generating drug-reactive T cells [26,27,28]. Interestingly, the conventional antigen-processing pathway normally required for the T-cell stimulation of proteins is bypassed in this model. The altered peptide repertoire model suggests that a drug (e.g., abacavir) binds non-covalently to the binding pocket of an HLA, altering its conformation and allowing a new array of self-peptides to occupy it stably and stimulate T cells [29,30]. Once T cells can be stimulated and activated by a drug through these mechanisms, the resultant effector immune mechanisms in turn contribute to the clinical manifestations of drug hypersensitivity.

### 3.2. Human Leukocyte Antigens (HLAs)

Genetic factors that influence immune responses and drug metabolism might confer susceptibility to drug hypersensitivity reactions. Pharmacogenomic studies conducted over the last decade have revealed that drug hypersensitivity reactions to several drugs are associated with specific HLA alleles. For example, associations have been reported to exist between HLA-B*57:01 and abacavir-induced hypersensitivity reactions [31,32], HLA-B*15:02 and carbamazepine-induced SJS/TEN [33], and HLA-B*58:01 and allopurinol-induced SCARs (SJS/TEN and DIHS/DRESS) [34]. Since then, a growing number of specific class I and/or class II HLA alleles have been reported to be associated with drug hypersensitivity reactions, and these associations are usually drug- and ethnicity-specific [5,23]. These pharmacogenomic discoveries have led to HLA-allele-specific screening for the prevention of serious reactions. Examples include screening for HLA-B*57:01 to avoid abacavir hypersensitivity reactions, which is routinely performed in human immunodeficiency virus (HIV) patients, and for HLA*B15:02 to avoid carbamazepine-associated SJS/TEN in Asian populations, which could reduce the occurrence of SCARs [5,23]. These findings imply that specific HLA molecules may have higher binding affinities for specific drug antigens and present the drug antigens to specific TCRs, causing a series of T-cell activation reactions and adverse immune responses. However, it has been suggested that HLA risk alleles are necessary, but not sufficient, for T-cell-mediated drug reactions because tolerant patients who carried HLA risk alleles have been reported [23,34]. Associations between DIHS/DRESS and particular HLAs have also been found, but no specific HLA alleles that are only associated with DIHS/DRESS have been reported. These findings suggest that some other factors, such as the underlying immune status of the patient, viral infections, and renal or hepatic insufficiency might determine whether patients develop DIHS/DRESS [4].

### 3.3. Viruses

#### 3.3.1. Roles of Herpesviruses in DIHS/DRESS

The mechanism underlying the paradoxical worsening and flaring-up of clinical symptoms after the discontinuation of the causative drug in DIHS/DRESS patients cannot be explained solely by an immunological reaction to the drug. It is now largely accepted that DIHS/DRESS can be associated with the reactivation of HHV-6, as HHV-6 DNA is detected in the peripheral blood of patients, and such findings have been used to diagnose DIHS [7]. Our finding that HHV-6-derived microRNAs, which regulate key viral genes, were detected in the sera and peripheral blood mononuclear cells (PBMCs) of patients with DIHS/DRESS also supports this [35]. However, the contribution of HHV-6 reactivation to the pathogenesis of DIHS/DRESS and the pathomechanism responsible for the visceral organ involvement observed in DIHS/DRESS remain unclear. The fact that an association was found between the detection of HHV-6 DNA and the flaring-up of symptoms, such as fever and hepatitis, indicates the relevance of HHV-6 reactivation to the flaring-up of clinical symptoms in DIHS/DRESS [36]. Furthermore, in previous studies HHV-6 DNA was detected in the skin [10] and lymph nodes [37] of DIHS/DRESS patients, suggesting that rashes and lymphadenopathy might be closely related to HHV-6 reactivation in DIHS/DRESS. The direct detection of HHV-6 in visceral organs has also been reported in DIHS/DRESS patients [38]. We previously reported the case of a patient with DIHS/DRESS, who developed acute renal failure and died from an opportunistic infection and multiorgan failure. At autopsy, we detected HHV-6 DNA in the kidney specimen, but not in the specimens from the other organs. The tubular epithelial cells in the kidney were also positively stained for the HHV-6 antigen [38]. Interestingly, CMV had never been detected in this patient, suggesting that HHV-6 alone can cause end-organ damage. Another report also described positive immunohistochemical HHV-6 antigen staining of the renal tubular epithelial cells of a renal biopsy specimen from a DIHS patient who developed renal dysfunction [39]. These findings suggest that renal dysfunction might be directly caused by HHV-6 reactivation in renal tissue. Taken together, HHV-6 reactivation might be associated with the pathogeneses of various clinical symptoms, such as organ failure, rashes, and lymphadenopathy, in patients with DIHS/DRESS. However, some investigators consider that a pathogenic role of HHV-6 reactivation in DIHS/DRESS is still controversial [40].

The reactivation of other herpesviruses, including HHV-7, Epstein-Barr virus (EBV) [41], and CMV [42], has also been reported to occur in DIHS/DRESS. Kano et al. demonstrated that human herpesviruses reactivate sequentially during the course of DIHS/DRESS. HHV-6 and/or EBV are the first to reactivate, followed by HHV-7 and eventually CMV, which is a similar sequential order to that seen in graft-versus-host disease [43]. The sequential reactivation of these herpesviruses may also explain the prolonged clinical symptoms and repeated exacerbation of clinical symptoms in different organs despite the discontinuation of the causative drug seen in DIHS/DRESS [7].

The definitions of CMV infection and disease are well-established, especially for immunocompromised patients in a transplant setting [44]. It is well known that CMV infection can cause characteristic CMV diseases, including pneumonia, gastrointestinal disease, hepatitis, retinitis, encephalitis/ventriculitis, nephritis, cystitis, myocarditis, and pancreatitis [44]. According to the developed definitions, the presence of appropriate clinical symptoms and/or signs together with the detection of CMV in tissue from the relevant organ based on a histopathological examination, virus isolation, rapid culturing, immunohistochemistry, and/or DNA hybridization are required to meet the criteria for “proven CMV end-organ disease” [44]. In this regard, some of the late-onset complications of DIHS/DRESS are undoubtedly caused by CMV, as they meet the criteria for CMV disease.

#### 3.3.2. Immunological Mechanism of Virus Reactivation

Despite the strong association between HHV-6 and DIHS/DRESS, the immunological mechanism underlying HHV-6 reactivation remains unknown. HHV-6 is known to exhibit selective tropism for CD4 T cells [45,46] and to latently infect monocytes/macrophages [47]. Recently, we found that CD134 was preferentially expressed on CD4 T cells in the acute stage of DIHS/DRESS, while this was not observed in other types of drug eruptions [48]. CD134, which is also called OX40, is a member of the tumor necrosis factor (TNF) receptor superfamily and is a well-characterized co-stimulatory receptor [49]. It was recently identified as a cellular receptor for HHV-6 [50]. We speculate that the upregulated expression of CD134 may contribute to the entry of HHV-6 into CD4 T cells in the acute stage of DIHS/DRESS. Furthermore, it has been demonstrated that the number of circulating monomyeloid precursors with the CD11b^+^CD13^+^CD14^−^CD16^high^ phenotype increased in the early stages of cases of DIHS harboring the HHV-6 antigen [51,52] and that these cells were able to transmit HHV-6 to skin-infiltrating CD4 T cells [52]. The results of these studies imply that monocytes/macrophages that have been latently infected with HHV-6 become reactivated during the early phase of DIHS/DRESS, leading to virus production, and the infectious viruses then infect CD4 T cells via CD134 and start replicating. However, this scenario only explains a part of the HHV-6 reactivation process, and the cause of such HHV-6 reactivation remains unknown. Takahashi et al. suggested that regulatory T cells (Tregs) make a critical contribution to the pathogenesis of DIHS/DRESS [53]. They demonstrated that a marked expansion of functional Tregs occurred in the acute stage of DIHS/DRESS and suggested that this may explain how herpesviruses are sequentially reactivated and why positive LTT reactions to causative drugs are not seen in the acute stage of the disease [4,53]. They also showed that Treg populations contracted upon the resolution of DIHS/DRESS, which might lead to an increased risk of subsequently developing autoimmune disease [4,53]. A further study examining the detailed mechanism responsible for these changes in the Treg population demonstrated that selective depletion of patrolling monocytes (pMOs) occurs in the acute stage of DIHS/DRESS, resulting in an increased frequency of classical monocytes, which could act to expand Tregs via the release of interleukin (IL)-10 [54]. After the resolution of DIHS/DRESS, pMOs were recruited, and the frequency of Th17 cells was significantly increased in response to interleukin (IL)-6 production by the pMOs [54]. It was suggested that the gradual shift from Tregs to Th17 cells observed during the clinical course of DIHS/DRESS, which is mediated based on the predominance of particular subsets of monocytes, could explain why a variety of autoimmune responses can develop over prolonged periods of time after the clinical resolution of DIHS/DRESS [4,54].

HHV-6 infections are frequently encountered in immunosuppressed patients, such as bone marrow transplant recipients and patients with acquired immunodeficiency syndrome (AIDS). In DIHS/DRESS, reductions in immunoglobulin levels and the B-cell count [55] as well as the expansion of Tregs [53] are observed in the acute stage and are considered to cause immunosuppression. The reactivation of HHV-6 could be facilitated by this immunosuppressed state and occurs as a transient event, i.e., it is detected at 2–3 weeks after onset in the vast majority of patients. Rarely, especially in severe cases, symptoms continue to deteriorate or several flare-ups are seen during the course of DIHS/DRESS. Although these disease courses can be partly explained by the sequential reactivation of herpesviruses [43], another possible explanation has been proposed by us, persistent HHV-6 reactivation [56]. We demonstrated that in some patients, HHV-6 DNA continued to be detected long after the onset of the disease and was sometimes associated with the frequent recurrence of clinical symptoms, such as rashes [56,57]. We also demonstrated that in DIHS/DRESS patients with persistent HHV-6 infections, CD4 T cells served as the main HHV-6 reservoir throughout the course of the disease, and low levels of an immediate early gene transcript (U90) were detected in CD4 T cells. These findings suggest that low-level HHV-6 reactivation persisted in these patients [56]. However, it is currently unclear whether DIHS/DRESS patients with persistent HHV-6 infections are in an immunosuppressed state throughout the course of their disease.

### 3.4. Effector T cells

As described above, most types of drug hypersensitivity, including DIHS/DRESS, involve T-cell-mediated immune responses against drugs. Drug-specific T cells have been identified in these conditions and are assumed to be the primary pathological effector cells. Pharmacogenomic studies have revealed that SCARs are predominately associated with HLA class I alleles although associations with HLA class II alleles have also been reported [5,23]. These pharmacogenomic discoveries indicate that CD8 T cells play a crucial role in the pathogenesis of SCARs. Indeed, laboratory evidence has shown that abacavir hypersensitivity syndrome (AHS) is HLA-B*57:01-restricted and mediated by drug-specific CD8 T cells [58]. However, in many types of drug hypersensitivity drug-specific CD4 and CD8 T cells can be detected, which recognize small chemicals via their αβ-TCRs in a major histocompatibility complex (MHC)-dependent manner [25,59]. Interestingly, the vast majority of drug-specific T cells express a particular TCRVβ, which is indicative of oligoclonality [25,60,61], while T cells from AHS patients display a polyclonal response involving the expression of a broad range of Vβ TCRs [29]. This implies that drugs act as superantigens in drug hypersensitivity [25].

In studies of DIHS/DRESS, in vitro causative drug stimulation induced CD4 T cells to proliferate [62], and drug-specific T-cell clones isolated from DIHS/DRESS patients primarily consisted of CD4 T cells [63,64,65,66] and occasional CD8 T cells [63]. In addition, drug-specific CD4 T-cell clones from a DRESS patient only expressed certain TCRVβs and required TCR-MHC class II engagement [63,64]. These CD4 T-cell clones were cytotoxic when incubated with the causative drug [63,64]. CD8 T cells are suggested to contribute to the pathogenesis of DIHS/DRESS, as the reactivation of herpesviruses is an integral component of the disease process of DIHS/DRESS, and MHC class I is associated with DIHS/DRESS [5] although no HLA alleles have been found to exhibit specific associations with DIHS/DRESS. A new insight into the roles of effector T cells in the complex pathogenesis of DIHS/DRESS was first proposed by Hashizume et al. Using T cells from DIHS patients that had experienced CMV reactivation, they demonstrated that CD4 T cells proliferated and produced IFN-γ in response to the causative drug in vitro and that circulating CD8 T cells expressed a limited number of TCRs, which recognized CMV-derived peptides, suggesting that the CD4 T cells were drug-reactive, whereas the CD8 T cells responded to CMV-derived peptides [62]. In support of this hypothesis, Picard et al. demonstrated that circulating CD8 T cells were activated in patients with DRESS and secreted large amounts of TNF-α and IFN-γ [67]. Expanded populations of CD8 T cells in the blood, skin, livers, and lungs of these patients shared the same TCR repertoire, which specifically recognized one of several EBV epitopes [67]. They suggested that the cutaneous and visceral symptoms of DRESS are mediated by activated CD8 T cells, which are largely directed against EBV [67]. Taken together, it appears that CD4 cells and CD8 T cells play distinct roles in DIHS/DRESS, i.e., drug-specific oligoclonal CD4 T cells are activated and initiate drug-induced immune responses, before CD8 T cells recognize viral antigens and initiate antivirus immune responses, which target multiple organs and amplify inflammatory responses.

Immune responses are often grouped into type 1, type 2, and type 3 responses. In DIHS/DRESS, eosinophilia [1,2], increased plasma IL-5 levels [68], and increased serum levels of Th2-associated chemokines, including thymus and activation-regulated chemokine (TARC) [69,70,71,72] and macrophage-derived chemokine (MDC) [71], have been detected in the acute stage. These findings suggest that the immune responses that occur at the onset of DIHS/DRESS are polarized toward type 2 immune responses. Indeed, the proportions of circulating IL-4- and IL-13-producing CD4 T cells (Th2 cells) were significantly higher in patients with DIHS/DRESS [73]. Furthermore, the upregulation of OX40L on PBMCs and CD134 (OX40) on CD4 T cells observed in the acute stage of DIHS/DRESS [74] also supports type 2 skewing because OX40-OX40L ligation is known to promote Th2 differentiation [75]. Collectively, these findings support the idea that the symptoms of DIHS/DRESS begin as an allergic reaction (a type 2 immune response) mediated by drug-specific CD4 T cells.

Finally, given the complex nature of the disease and its high mortality rate, the accurate diagnosis and management of DIHS/DRESS are necessary. Therefore, diagnostic markers for the disease urgently need to be identified. DIHS/DRESS can be difficult to diagnose because DIHS/DRESS often initially presents as a morbilliform eruption that is indistinguishable from other types of drug eruptions, and the reported symptoms mimic those of several other diseases, including infectious diseases, and can appear a long time after the initial exposure to the causative drug. Therefore, we wish to emphasize that a series of our studies have suggested that the serum levels of TARC [57,69,70,71,72] and MDC [71], and the extent of CD134 expression on CD4 T cells [48,74] may serve as useful biomarkers for the early diagnosis of DIHS/DRESS. A clinical trial to validate the effectiveness of using serum TARC levels for distinguishing DIHS/DRESS from other types of drug eruptions is currently underway in Japan.

## 4. Conclusions

Although great advances in our understanding of the pathophysiology of DIHS/DRESS have occurred during the last decade, DIHS/DRESS is still challenging to diagnose because its diagnosis can be delayed, or the disease can go unrecognized as a drug-related condition because of its relatively late onset; gradual evolution; persistence even after the cessation of treatment with the causative drug; and the variations in its presentation, course, and severity. In addition, dissecting the pathophysiology of DIHS/DRESS also remains challenging due to the condition’s rarity, complexity, and variability. Further investigation of the HLA risk alleles for DIHS/DRESS, precisely how drugs activate T cells, and what triggers herpesvirus reactivation and the development of autoimmune diseases is required. Addressing these issues might eventually facilitate the preclinical prediction and prevention of DIHS/DRESS.

## Figures and Tables

**Table 1:** Diagnostic criteria for drug-induced hypersensitivity syndrome (DIHS) established by a Japanese consensus group [1].

1	Maculopapular rash developing > 3 weeks after starting with a limited number of drugs
2	Prolonged clinical symptoms 2 weeks after discontinuation of the causative drug
3	Fever (>38 °C)
4	Liver abnormalities (alanine aminotransferase > 100 U·L^−1^) ^a^
5	Leukocyte abnormalities (at least one present)
a	Leukocytosis (>11 × 10^9^ L^−1^)
b	Atypical lymphocytosis (>5%)
c	Eosinophilia (>1.5 × 10^9^ L^−1^)
6	Lymphadenopathy
7	Human herpesvirus 6 reactivation

The diagnosis is confirmed by the presence of the seven criteria above (typical DIHS) or of the five (1~5) (atypical DIHS). ^a^ This can be replaced by other organ involvement, such as renal involvement.

**Table 2:** Inclusion criteria for potential case of HSS/DRESS in RegiSCAR [2].

Hospitalization
Reaction suspected to be drug related
Acute skin rash ^a^
Fever above 38 °C ^a^
Enlarged lymph nodes at at least two sites ^a^
Involvement of at least one internal organ ^a^
Blood count abnormalities
Lymphocytes above or below the laboratory limits ^a^
Eosinophils above the laboratory limits (in percentage or absolute count) ^a^
Platelets below the laboratory limits ^a^

^a^ Three or more required.
